# The Novel Autophagy Inhibitor Alpha-Hederin Promoted Paclitaxel Cytotoxicity by Increasing Reactive Oxygen Species Accumulation in Non-Small Cell Lung Cancer Cells

**DOI:** 10.3390/ijms19103221

**Published:** 2018-10-18

**Authors:** Yujuan Zhan, Kun Wang, Qiao Li, Yidan Zou, Bonan Chen, Qing Gong, Hiuting Idy HO, Ting Yin, Fangyuan Zhang, Yuhua Lu, Weijie Wu, Yilin Zhang, Yuhui Tan, Biaoyan Du, Xiaodong Liu, Jianyong Xiao

**Affiliations:** 1 Department of Biochemistry, Guangzhou University of Chinese Medicine, Guangzhou 510006, China; 20161104381@stu.gzucm.edu.cn (Y.Z.); 20171104455@stu.gzucm.edu.cn (B.C.); 2016101053@stu.gzucm.edu.cn (T.Y.); 2016101035@stu.gzucm.edu.cn (W.W.); tyuhui@gzucm.edu.cn (Y.T.); 2 Research Center for Integrative Medicine, Guangzhou University of Chinese Medicine, Guangzhou 510006, China; 20172104085@stu.gzucm.edu.cn; 3 Department of Pathology, Guangzhou University of Chinese Medicine, Guangzhou 510006, China; 2016102051@stu.gzucm.edu.cn (F.Z.); 2017102022@stu.gzucm.edu.cn (Y.L.); 2017102042@stu.gzucm.edu.cn (Y.Z.); dubiaoyan@gzucm.edu.cn (B.D.); 4 School of Pharmaceutical Science, Guangzhou University of Chinese Medicine, Guangzhou 510006, China; 2305455396lq@gmail.com; 5 Department of Anaesthesia and Intensive Care, The Chinese University of Hong Kong, Hong Kong SAR 999077, China; 1155119213@link.cuhk.edu.hk (Y.Z.); b144156@cuhk.edu.hk (H.I.H.); 6 GMU-GIBH Joint School of Life Sciences, Guangzhou Medical University, Guangzhou 511436, China; 2010990020@gzhmu.edu.cn

**Keywords:** alpha-hederin, autophagy inhibition, non-small cell lung cancer, chemotherapy, paclitaxel, synergistic effect

## Abstract

Chemoresistance is a major limiting factor that impairs the outcome of non-small cell lung cancer (NSCLC) chemotherapy. Paclitaxel (Tax) induces protective autophagy in NSCLC cells, leading to the development of drug resistance. We recently identified a new autophagy inhibitor (alpha-hederin) and hypothesized that it may promote the killing effect of Tax on NSCLC cells. We found that alpha-hederin (α-Hed) could block late autophagic flux in NSCLC cells by altering lysosomal pH and inhibiting lysosomal cathepsin D maturation. Combination treatment of α-Hed and Tax synergistically reduced NSCLC cell proliferation and increased NSCLC cell apoptosis compared with treatment with α-Hed or Tax alone. Furthermore, α-Hed plus Tax enhanced the accumulation of intracellular reactive oxygen species (ROS) in NSCLC cells, while the ROS inhibitor N-acetylcysteine reversed the inhibitory effect of the combination treatment. Our findings suggest that α-Hed can increase the killing effect of Tax on NSCLC cells by promoting ROS accumulation, and that combining α-Hed with classical Tax represents a novel strategy for treating NSCLC.

## 1. Introduction

Lung cancer is the most prevalent cause of cancer-related death in men and women around the world; non-small cell lung cancer (NSCLC) is the most common type, with high morbidity and mortality [1]. To date, the main strategy in cancer therapy consists of surgery, radiotherapy, and chemotherapy. Surgery and radiotherapy are typically used to remove tumor cell foci [2], while chemotherapy is mainly used as a postoperative adjuvant treatment in clinical practice because chemotherapy drugs can generally spread throughout the body and kill tumor cells effectively [3]. For patients with advanced tumors, even though it is difficult to eradicate cancer cells, chemotherapy can alleviate suffering and improve quality of life [4]. Nonetheless, NSCLC cell chemoresistance due to long-duration drug therapy has become a major obstacle that limits chemotherapy outcomes.

Factors such as increased drug efflux pump expression [5], mutation of tubulin, and increased βIII tubulin isomer expression [6] contribute to the generation of NSCLC chemoresistance. Notably, autophagy is an important mechanism of chemoresistance, because it can increase NSCLC cell survival ability under stress [7].

Autophagy is an evolutionarily conserved process where an autophagosome (a double-membrane vacuole) envelops the cytoplasmic constituents of a cell and delivers them to the lysosome for degradation [8]. In addition to its role in waste protein and organelle turnover, autophagy has several pathological and physiological roles [9,10]. Moreover, it plays a fairly vital role in numerous human diseases, including cancer [11,12].

Autophagy is induced when cells encounter environmental stressors such as pathogen infection and nutrient starvation, providing the nutrients and energy required for cell survival. Accordingly, autophagy has been acknowledged as being cytoprotective against environmental stress [13,14]. In the later stages of cancer, tumor cells frequently experience environmental stresses such as lack of nutrition, hypoxia, or chemotherapy-induced cytotoxicity, but autophagy cannot only help cancer cells survive, but can also induce chemoresistance. Therefore, inhibiting cancer cell autophagy is likely, on one hand, to repress growth; on the other hand, it can alleviate chemotherapy resistance. To date, the inhibitors of autophagy—chloroquine (CQ) and its derivative hydroxychloroquine (HCQ)—have been used for treating patients with malignant tumor. The clinical outcomes confirm the viability and advantageous effects of using CQ or HCQ in combination or alone in cancer therapy [15,16,17].

Paclitaxel (Tax) is a first-line chemotherapeutic drug for treating patients with NSCLC. High Tax concentrations can bind tubulin polymer, stabilizing the spindle microtubules and preventing their disassembly in vivo, thereby arresting tumor cells in G2/M and ultimately leading to apoptosis [18]. However, low Tax concentrations stabilize the interphase microtubule, causing G0/G1 arrest, which may not cause cell death because the repair mechanism is activated [19]. Tax can induce protective autophagy in NSCLC cells, leading to the development of drug resistance that can be reversed by autophagy inhibition [20].

**Figure 1:**
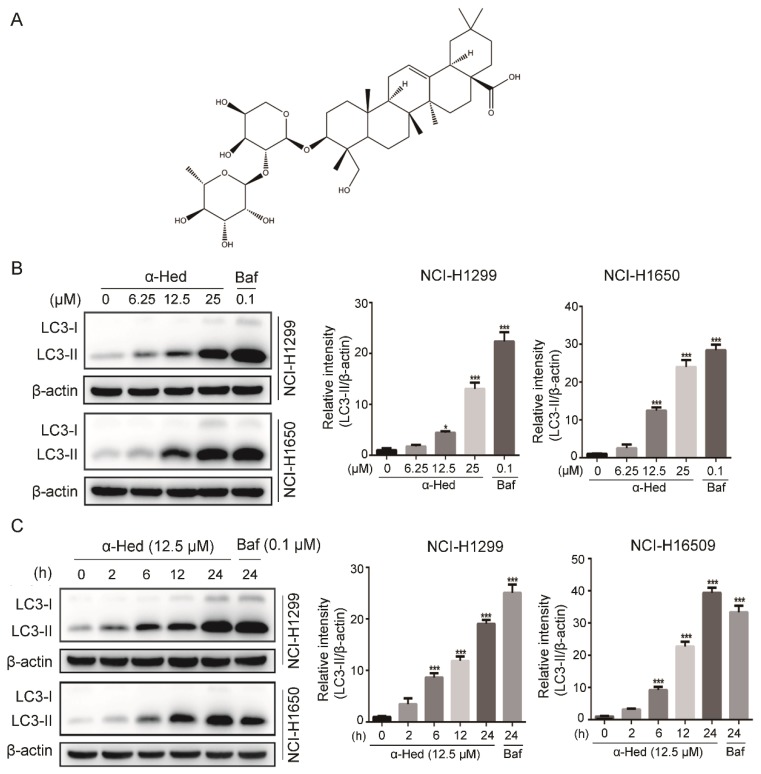

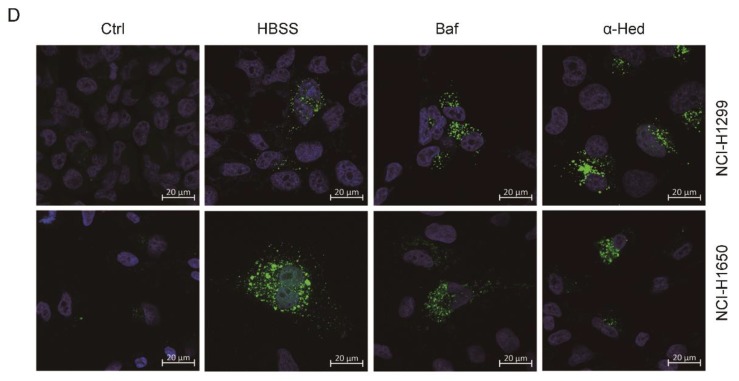
Alpha-hederin (α-Hed) induced the increased number of autophagosomes in human non-small cell lung cancer (NSCLC) cells. (**A**) The chemical structure of α-Hed. (**B**,**C**) α-Hed promoted microtubule-associated protein light chain 3 (LC3)-II accumulation in a dose- and time-dependent manner. NCI-H1299 and NCI-H1650 cells were treated with α-Hed (12.5 μM) or bafilomycin A1(Baf, 0.1 μM) for the indicated time courses (**C**), or treated with α-Hed or Baf at the indicated doses for 24 h (**B**). LC3-II was quantified and the fold increase is presented. *** *p* < 0.001. (**D**) Effect of α-Hed on green fluorescent protein (GFP)-LC3 punctation. NCI-H1299 and NCI-H1650 cells were transiently transfected with GFP-LC3 plasmid, α-Hed (12.5 μM), Baf (0.1 μM) for 24 h, or with Hank’s balanced salt solution (HBSS) for 6 h. Typical images are shown.

Natural products are an important source of lead compounds and play an important role in drug discovery. Recently, we identified a novel autophagy inhibitor, alpha-hederin (α-Hed), by functional screening of compounds in the UNPD (Universal Natural Products Database) natural product library [21]. α-Hed, a major saponin (Figure 1A) extracted from *Hedera helix*, had wide biological activities, including anti-oxidation, anti-inflammation, and anti-diabetes [22,23,24]. To date, several studies have proposed that α-Hed shows a certain degree of anti-cancer activity. For example, it inhibited interleukin 6–induced epithelial–mesenchymal transition in colon cancer cells [25] and induced apoptosis in hepatocellular carcinoma cells [26].

In the present study, we determined that α-Hed could inhibit autophagy in NSCLC cells by altering the lysosomal pH and inhibiting lysosomal cathepsin maturation. Further data indicated that α-Hed synergized with Tax to induce human NSCLC cell apoptosis in a caspase-3–dependent manner. Although it is widely accepted that autophagy inhibition with small molecule inhibitors is a promising cancer therapeutic strategy, effective and specific autophagy inhibitors are rare. We anticipate that our findings can provide a theoretical foundation for α-Hed as a novel candidate in combination treatment of NSCLC.

## 2. Results

### 2.1. α-Hed Induced the Increased Autophagosome Numbers in Human NSCLC Cells

During autophagy, ATG4 cleaves LC3B (microtubule-associated protein light chain 3B) to generate the cytoplasmic form LC3-I, which can be further modified and converted to the phagophore-associated LC3-II through conjugation with the lipid phosphatidylethanolamine [27]. Accordingly, LC3-II expression levels are correlated well with the number of autophagosomes. To determine whether α-Hed affected the autophagic process of NSCLC cells, we first examined LC3-II expression levels in NSCLC cell lines (NCI-H1299, NCI-H1650) after α-Hed or Baf (positive control) treatment. Western blotting showed that α-Hed caused LC3B-II accumulation of in NSCLC cells in a dose- and time-dependent manner (Figure 1B,C).

To confirm the effect of α-Hed on NSCLC cell autophagy, we examined LC3 protein expression and localization. NSCLC cells were transiently transfected with green fluorescent protein (GFP)-LC3 plasmid, a canonical autophagosome marker with green fluorescence. The number of GFP-LC3 puncta (green fluorescence) is usually used to measure the autophagosome numbers [28]. Figure 1D shows that α-Hed increased GFP-LC3 puncta formation dramatically, which was consistent with that of the positive controls (HBSS [Hanks’ balanced salt solution] and Baf treatment). These data indicated that α-Hed treatment caused the increased number of autophagosomes in human NSCLC cells.

### 2.2. α-Hed Inhibited NSCLC Cell Autophagic Flux

**Figure 2:**
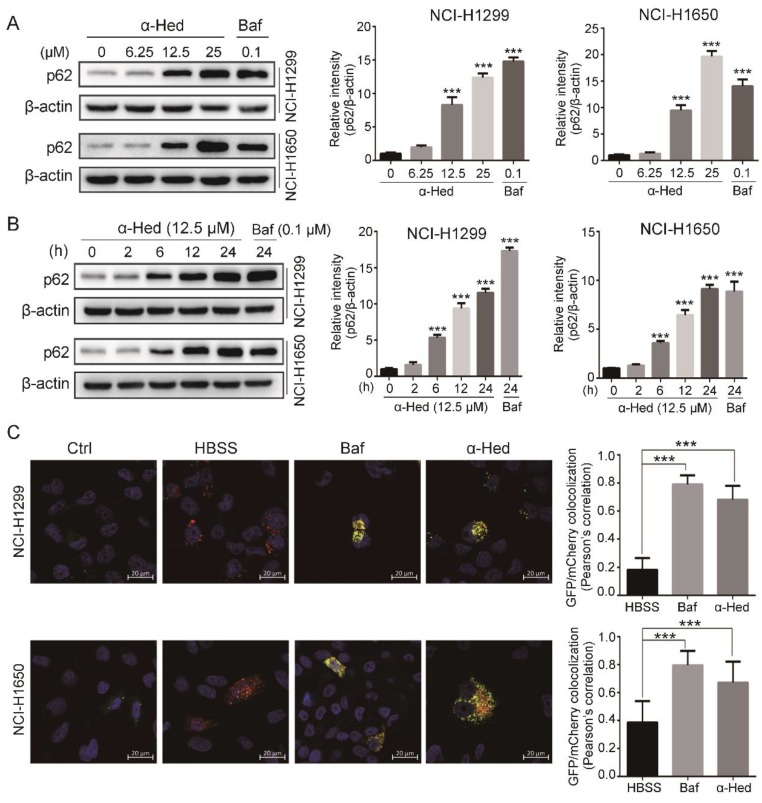
α-Hed inhibited autophagic flux in human NSCLC cells. (**A**,**B**) α-Hed promoted p62 accumulation in a dose- and time-dependent manner. NCI-H1299 and NCI-H1650 cells were treated with α-Hed (12.5 μM) or Baf (0.1 μM) for the indicated time courses (**B**), or treated with α-Hed or Baf at the indicated doses for 24 h (**A**). p62 was quantified and the fold increase is presented. *** *p* < 0.001. (**C**) Cells were transiently transfected with mCherry-GFP-LC3 plasmid and treated with vehicle, α-Hed (12.5 μM), Baf (0.1 μM) for 24 h, or HBSS for 6 h. Typical images are shown. *** *p* < 0.001.

Elevated LC3-II protein levels or GFP-LC3 puncta formation may represent increased autophagosome generation (promotion of autophagic flux) or autophagosomal maturation and cargo degradation blockade (inhibition of late autophagic flux) [28,29]. Therefore, we attempted to clarify whether α-Hed inhibited or promoted NSCLC cell autophagic flux by testing p62 (SQSTM1) expression levels. p62 can be selectively incorporated into the autophagosome through direct binding to LC3; typically, it is subsequently degraded by autophagy [30]. When late autophagic flux is impaired, p62/SQSTM1 cannot be degraded normally, and its levels eventually increase. Consequently, increased p62 expression levels are commonly used to indicate autophagic flux inhibition [28]. In the present study, α-Hed upregulated p62 in the NSCLC cells time- and dose-dependently (Figure 2A,B), suggesting that α-Hed is probably an autophagy inhibitor.

To verify the inhibitory effect of α-Hed on autophagic flux in NSCLC cells, we transfected the cells with a tandem reporter plasmid expressing mCherry-GFP-LC3 fusion protein and treated the cells with α-Hed (12.5 μM), Baf (0.1 μM; autophagic flux inhibitor; 24 h), or HBSS (autophagic flux inducer; 6 h). GFP-generated green fluorescence is readily quenched in acidic conditions such as the autolysosome, while the red fluorescence of mCherry is more stable under acidic environments [31]. We observed many red puncta in the HBSS-treated cells due to the quenching of green fluorescence in the acidic autolysosome. By contrast, autophagic flux suppression by Baf resulted in a large proportion of yellow puncta due to the merging of green and red fluorescence [28].

Consistent with Baf treatment, α-Hed treatment resulted in a large quantity of yellow puncta (green and red fluorescence overlay) (Figure 2C). Pearson’s correlation statistics of the red–green fluorescence colocalization also showed that the α-Hed- and Baf-treated cells had significantly higher colocalization ratios than HBSS-treated cells. These results suggest that α-Hed inhibits late autophagy, resulting in marked autophagosome accumulation.

### 2.3. α-Hed Altered the Lysosomal pH and Inhibited Lysosomal Cathepsin Maturation

In late autophagy, autophagosomes fuse with lysosomes to form autolysosomes, and then degrade their cargo. If lysosomal function is impaired, the cargo will not be properly degraded and autophagic flux will be inhibited. Decreasing lysosomal function by disrupting lysosomal cysteine protease maturation, mainly referring to cathepsin D (CatD) or lysosomal pH alteration, will inhibit late autophagic flux [28].

**Figure 3:**
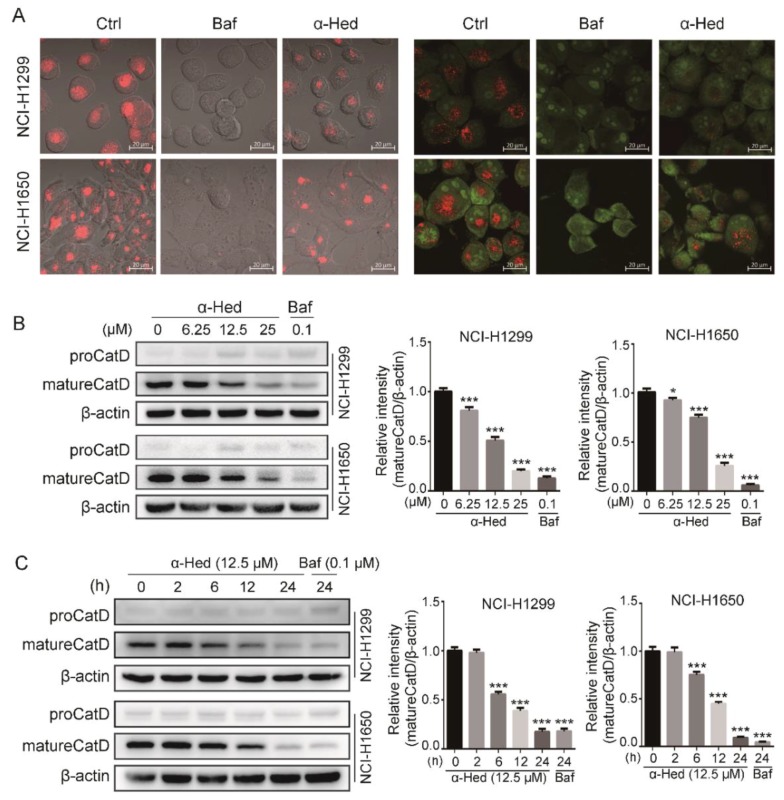
α-Hed altered the lysosomal pH and inhibited lysosomal cathepsin maturation. (**A**) α-Hed altered the lysosomal pH. Cells were treated with vehicle, α-Hed (12.5 μM), or Baf (0.1 μM) for 24 h. (**B**,**C**) α-Hed promoted mature cathepsin D (CatD) accumulation in a dose- and time-dependent manner. NCI-H1299 and NCI-H1650 cells were treated with α-Hed (12.5 μM) or Baf (0.1 μM) for the indicated time courses (**C**), or treated with α-Hed or Baf at the indicated doses for 24 h (**B**). Mature CatD was quantified and the fold increase is presented. * *p* < 0.05, *** *p* < 0.001.

To determine if α-Hed affected lysosomal acidity, LysoTracker Red and acridine orange (AO) were used to monitor the alteration of lysosomal pH. Bafilomycin A1 (Baf), which affects lysosomal pH [32], was used as a positive control. Compared with the negative control, α-Hed decreased the fluorescence intensity of LysoTracker Red and AO remarkably (Figure 3A). These data suggest that α-Hed affects lysosomal pH in NSCLC cells.

Most CatD is transferred into the lysosomes for maturation; lysosomal cathepsin maturation is essential for autophagic cargo degradation [33]. Mature CatD levels in NSCLC cells were examined by western blotting. α-Hed dose-dependently decreased mature CatD levels compared with the negative control (Figure 3B,C). These data demonstrate that α-Hed suppresses late autophagy by altering lysosomal pH and inhibiting lysosomal CatD maturation.

### 2.4. NSCLC Cell Tax Resistance Was Associated with Induction of Autophagy

**Figure 4:**
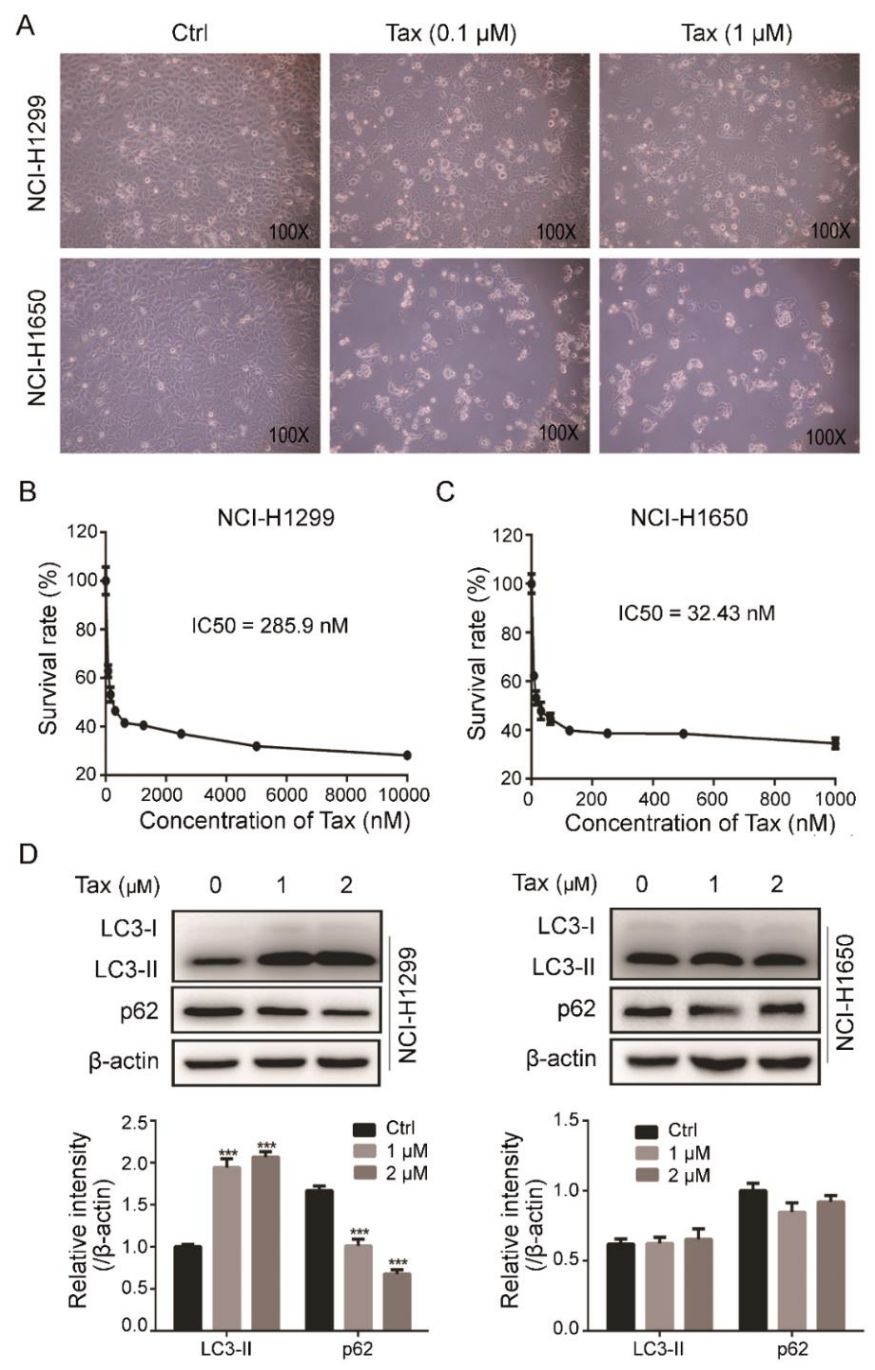
Paclitaxel (Tax) exerted different killing effects on different NSCLC cell lines, which was related to the degree of autophagy induction. (**A**) Cell morphology after 24-h treatment with 0.1 μM or 1 μM Tax. (**B**,**C**) Cell Counting Kit-8 (CCK-8) determination of viability of Tax-treated NCI-H1299 (**D**) and NCI-H1650 (**D**) cells. The median inhibitory concentration (IC50) was estimated by log(inhibitor) vs. normalized response nonlinear fit (GraphPad Prism 6.0). (**D**) Western blot evaluation of LC3 and p62 proteins in cells treated with Tax for 24 h. *** *p* < 0.001.

As autophagy inhibition can promote Tax-induced apoptosis in NSCLC cells [20], we hypothesized that α-Hed may synergize with Tax to kill NSCLC cells. First, the anti-cancer effect of Tax on the NSCLC cell lines NCI-H1299 and NCI-H1650 was examined by light microscopy photography. NCI-H1650 cells were much more sensitive to Tax than NCI-H1299 cells (Figure 4A). Furthermore, Cell Counting Kit-8 (CCK-8) indicated that the Tax median inhibitory concentration (IC50) for the NCI-H1299 cells was 285.9 nM, which was nearly nine times higher than that for the NCI-H1650 cells (32.4 nM) (Figure 4B,C).

Tax can induce protective autophagy and lead to chemoresistance in cancer cells [34]; therefore, Tax may hypothetically have differential autophagic effects on the NCI-H1299 and NCI-H1650 cell. Consistent with this, western blotting showed that LC3-II protein expression was dramatically elevated while p62 decreased in Tax-treated NCI-H1299 cells, but that in the NCI-H1650 cells was not significantly altered (Figure 4D). These data suggest that the NCI-H1299 cell chemoresistance was probably associated with the extent of cytoprotective autophagy induction. In subsequent experiments, we used the NCI-H1299 cell line to study the synergistic anti-tumor effect of α-Hed and Tax on NSCLC cells.

### 2.5. α-Hed Increased the Tax-Induced Inhibition of Proliferation and Apoptosis in NCI-H1299 Cells

**Figure 5:**
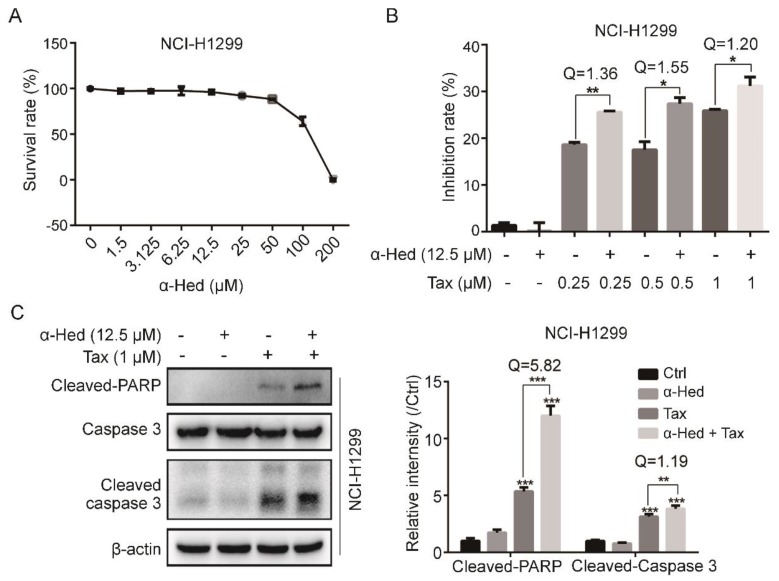
α-Hed increased the Tax-induced inhibition of proliferation and apoptosis in human NSCLC cells. (**A**) Effect of α-Hed on the NCI-H1299 cell survival rate. Cells were treated by α-Hed for 24 h. (**B**) α-Hed increased Tax-induced proliferative inhibition. * *p* < 0.05, ** *p* < 0.01. *Q* value > 1.15 indicted a synergistic effect. (**C**) α-Hed increased Tax-induced apoptosis. Cleaved-PARP, caspase 3, and Cleaved-caspase 3 expression levels were detected with western blotting. ** *p* < 0.01, *** *p* < 0.001. *Q* value > 1.15 indicates a synergistic effect.

To optimize the concentration of α-Hed for preparing the combination treatment, we used CCK-8 to examine the cytotoxicity of α-Hed treatment alone on NCI-H1299 cells. Figure 5A shows that, at concentrations of less than 50 µM, α-Hed had mild cytotoxic effects. Referring to the α-Hed concentration in autophagy inhibition, we used 12.5 µM α-Hed for the subsequent experiments.

We used CCK-8 determine whether α-Hed increased the anti-cancer effect of Tax. Figure 5B shows that the α-Hed plus Tax combination treatment had a significantly increased rate of inhibition as compared with Tax alone. The *Q* value (>1.15) indicated synergy between α-Hed and Tax. Furthermore, the increased levels of cleaved poly (ADP-ribose) polymerase (PARP) and cleaved-caspase 3 expression in the NCI-H1299 cells following the combination treatment suggested that α-Hed might promote caspase-dependent cell death in chemoresistant human NSCLC cells (Figure 5C).

### 2.6. α-Hed Sensitized NCI-H1299 Cells to Tax by Promoting Reactive Oxygen Species (ROS) Accumulation

Tax could lead to ROS production in cancer cells [35], and excessive ROS accumulation causes cell death [36]. As autophagy is an important ROS scavenger [37], inhibition of autophagy is likely to lead to excessive ROS accumulation in Tax-treated cancer cells, consequently enhancing its killing effect on tumor cells.

**Figure 6:**
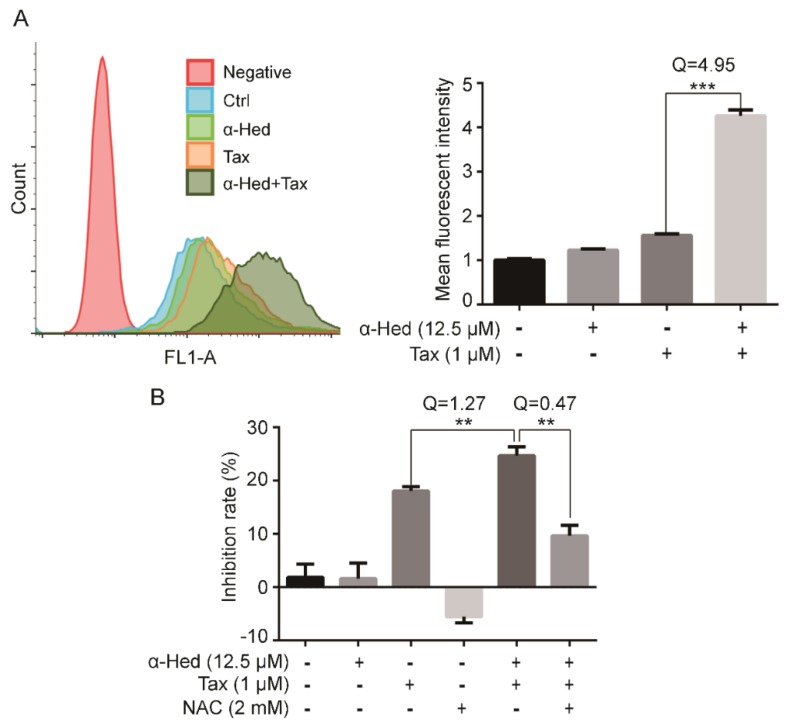
α-Hed sensitized cells to Tax-induced cell death via excessive reactive oxygen species (ROS) accumulation. (**A**) α-Hed pretreatment significantly increased ROS accumulation. *** *p* < 0.001. *Q* value > 1.15 indicated a synergistic effect. (**B**) N-acetylcysteine (NAC) reversed Tax-induced inhibition of cell proliferation. Cells were treated with or without α-Hed or Tax; 24 h later, the survival rate was detected using CCK-8. ** *p* < 0.01, *** *p* < 0.001. *Q* value > 1.15 indicates a synergistic effect; *Q* value < 0.85 indicates an antagonistic effect.

We used the green fluorescent probe DCFDA (2′,7′-dichlorofluorescein diacetate) and flow cytometry to measure the amount of intracellular ROS. Following treatment with α-Hed or Tax alone, only a small amount of ROS was detected in the NCI-H1299 cells (Figure 6A). By contrast, α-Hed plus Tax treatment significantly upregulated ROS in the cells. These data suggest that α-Hed promotes Tax-induced ROS accumulation.

Next, we inhibited ROS in the cells treated with α-Hed plus Tax using N-acetylcysteine (NAC), a classic ROS inhibitor, and found that the inhibition rate of NCI-H1299 cells significantly decreased compared with α-Hed and Tax treatment (Figure 6B). These data suggest that α-Hed promotes Tax-induced cell death via excessive ROS accumulation.

## 3. Discussion

The regulation of autophagy may have two diametrically opposite effects in tumor development. First, as a cell survival pathway, whether basal or activated by stress, autophagy can prevent the accretion of oncogenic proteins, damaged proteins, and damaged organelles, thereby preventing long-term tissue damage and cell death; in this case, autophagy can reduce the risk of tumorigenesis [38]. In addition, autophagy can yield metabolic substrates through autophagy-mediated intracellular material circulation after tumor formation, maintain important mitochondrial functions, and thereby promote tumor growth [38].

Therefore, in early tumor development, chronic tissue damage, inflammation, and genomic instability can produce a microenvironment conducive to tumorigenesis and tumor development [39,40]. Subsequently, autophagy activation can eliminate carcinogenic factors such as oncogenic proteins, thereby inhibiting tumorigenesis.

When a tumor develops to a certain stage, the tumor cells will face survival pressures such as hunger and local anemia (glucose deficiency); autophagy can restore tumor cell growth and metabolism by reusing substances, quality control of proteins and organelles, maintaining amino acid levels, upregulating starvation-related genes, and enhancing mitochondrial function. These processes can help tumor cells withstand survival pressure [41,42,43]. Therefore, in autophagy-based treatments, individualized treatment strategies should be used for different stages of tumor development in different patients. Indeed, upon diagnosis, the vast majority of patients with cancer would have passed the initial stage in which autophagy can inhibit tumorigenesis. Therefore, autophagy suppression can be a very promising therapeutic strategy. Additionally, CQ and HCQ have been used as autophagic inhibitors; their feasibility and beneficial effects have been demonstrated recently [15,16,17].

Here, we provided for the first time clear evidence that α-Hed induces autophagosome accumulation by altering the lysosomal pH and inhibiting lysosomal cathepsin maturation, as evidenced by the following: (i) α-Hed treatment resulted in an increased number of autophagosomes in human NSCLC cells (Figure 1); (ii) α-Hed inhibited human NSCLC cell autophagic flux (Figure 2); (iii) α-Hed altered the lysosomal pH markedly, attenuating the enzymatic maturation of lysosomal cathepsins (Figure 3). CatD is involved in numerous pathologies and oncogenic processes in cancer [44], inhibiting CatD activity represents a promising cancer therapeutic strategy. As the vacuolar ATPase (v-ATPase), a proton pump plays a major role in maintaining the low acidic environment (around 4.5) in lysosomal lumens [45], hypothetically, α-Hed impinged on the acidification of lysosome maybe through negatively regulating v-ATPase. In addition, although the Ca^2+^/H+ exchanger was not found in placental mammals, Ca^2+^ uptake and maintenance depend on the acidic pH in lysosome [46]. A tantalizing question arises as to whether cellular Ca^2+^ concentration has an impact on the lysomal H+ gradient. The possible mechanisms by which α-Hed inhibited the acidity of lysosome need more investigation. In addition, compounds such as CQ and Baf, which can affect lysosome acidification, can also affect autophagosome and lysosome fusion [47]. Therefore, we have reason to speculate that α-Hed may also interfere with autophagosome and lysosome fusion, which warrants further exploration.

Interestingly, we also found that Tax had a heretofore very different inhibitory effect on two NSCLC cell lines. The NCI-H1650 cell line was very sensitive to Tax and NCI-H1299 was more resistant to Tax; the IC50 were 32.43 nM (NCI-H1650) vs. 285.9 nM (NCI-H1299) (Figure 4B,C). Then, we examined the induction of autophagy in the two cell lines at the same concentration of Tax, and found that the NCI-H1299 cells had significantly increased autophagy levels, whereas that of the NCI-H1650 cells was not significantly changed (Figure 4D). This suggests that Tax sensitivity of these two cell lines is likely related to the degree of autophagy induction. Accordingly, NCI-H1299 can be a naturally resistant cell line that would be appropriate for studying chemotherapy sensitization therapy. Subsequently, we studied the synergistic effect of α-Hed on Tax in the NCI-H1299 cell line. α-Hed increased the Tax-induced inhibition of proliferation and apoptosis in the cells (Figure 5). Additionally, we also found that α-Hed increased the cisplatin-induced inhibition of human NSCLC cell proliferation, where further investigation is required to reveal the underlying mechanism (Supplementary Materials).

As Tax can damage tumor cell mitochondria and lead to ROS production, and inhibiting autophagy can lead to the inability to clear damaged mitochondria, resulting in excessive ROS accumulation, which will in turn activate the death-related pathways, leading to increased cell death [48], we had reason to speculate that α-Hed exerted its synergistic effect on Tax through such a pathway, and Figure 6 proves us correct. Therefore, we may conclude that α-Hed sensitizes NSCLC cells to Tax-induced cell death via excessive ROS accumulation.

## 4. Methods and Materials

### 4.1. Chemicals and Reagents

α-Hed(A0742) and Tax(A0177) were purchased from Chengdu Must Bio-Technology (Chengdu, China); Dulbecco’s modified Eagle’s medium (DMEM), streptomycin, penicillin, HBSS, and fetal bovine serum (FBS) were from Gibco Life Technologies (Grand Island, NY, USA); Acridine Orange (AO, A6014), and Baf (B1793) were from Sigma Biotechnology (St. Louis, MO, USA); LysoTracker Red DND-99 (L7528) was from Invitrogen (Carlsbad, CA, USA); anti-LC3B antibody (12994S), anti-p62 antibody (5114), anti-PARP antibody (9542), and cleaved-caspase 3 antibody (9662) were from Cell Signaling Technology (Boston, MA, USA); CatD antibody (SC-13985) was from Santa Cruz Biotechnology (Dallas, TX, USA). Peroxidase-labeled antibody to mouse IgG (AS003) and peroxidase-labeled antibody to rabbit IgG (AS014) were from ABclonal (Wuhan, China).

The GFP-LC3B and mCherry-GFP-LC3B plasmids were gifts from professor William KK Wu (The Chinese University of Hong Kong).

### 4.2. Cell Culture

The human NSCLC cell lines NCI-H1299 (ATCC^®^ CRL-5803™) and NCI-H1650 (ATCC^®^ CRL-5883™) were obtained from American Type Culture Collection (Rockville, MD, USA). All the cells were cultured in DMEM supplemented with 10% FBS and penicillin (100 U/mL)/streptomycin (100 U/mL) at 37 °C in a humidified atmosphere with 5% CO_2_.

### 4.3. Cell Viability Assay

We plated 5000 cells per well in 96-well plates and cultured them for 24 h at 37 °C in 5% CO_2_. The cells were treated with different compounds for 24 h. Then, the medium was removed, and then 100 µl CCK-8 reagent diluted 10 times with DMEM was added to each well and allowed to incubate for 2 h in a humidified atmosphere of 5% CO_2_ at 37 °C. The absorbance of each well was measured at 450 nm. Cell proliferation was assessed using CCK-8 according to the manufacturer’s protocol (Dojindo Laboratories, Kumamoto, Japan).

### 4.4. GFP-LC3B or mCherry-GFP-LC3B Assay

Cells (8 × 104/well) were seeded on coverslips and cultured in 12-well plates for 24 h, and then transfected with GFP-LC3B or mCherry-GFP-LC3B plasmids using Lipofectamine 3000 strictly according to the manufacturer’s instructions (Invitrogen). After 6 h transfection, the culture medium was replaced with basic DMEM. After 24 h, the cells were incubated with the drugs or starved. Then, the cells were fixed with 4% paraformaldehyde for 20 min, washed three times with phosphate-buffered saline (PBS), incubated with Hoechst 333,428 (Beyotime Biotechnology, Haimen, China) in PBS for 10 min, and mounted on a glass slide using Prolong Diamond DAPI (P36966, Karlsruhe, Germany). Images were captured using an LSM 800 confocal microscope (Carl Zeiss, Jena, Germany). For quantification, we randomly chose >20 cells (per experiment), and quantified the colocalization of green and red fluorescence using a colocalization tool in ImageJ (NIH, Bethesda, MD, USA).

### 4.5. Western Blotting

The cells were lysed in 1× loading buffer. Then, the samples were separated by 10% or 15% sodium dodecyl sulfate–polyacrylamide gel electrophoresis (SDS-PAGE) and transferred to nitrocellulose membranes. The membranes were blocked for 2 h with 5% skim milk and incubated overnight with each primary antibody at 4 °C. Then, the membrane was washed thrice with TBST (0.05% Tween 20 in Tris-buffered saline) every 5 min and incubated with the secondary antibodies (diluted 1:3000) as appropriate. We visualized the immunoreactive bands with enhanced chemiluminescence (ECL) using an ECL detection system. The band density was quantified using ImageJ (NIH).

### 4.6. AO Staining

Equal numbers of cells (8 × 104/well) were seeded on glass slides in 12-well plates for 24 h at 37 °C in 5% CO_2_. Then, the cells were incubated for 24 h with the indicated compound. After 24 h, the culture medium was removed, and the cells were washed three times with PBS before being stained with AO (5 μg/mL) (37 °C in 5% CO_2_). After 20 min, the cells were washed three times with PBS, and were observed under a laser confocal scanning microscope equipped with an argon laser (excitation wavelength 488 nm) and a 63× objective lens, and images were obtained. In the lysosomal compartments, AO produces red fluorescence (emission filter 620 nm long pass) and produces green fluorescence (emission between 520 and 560 nm) in the cytosol and nuclear compartments. The red and green intensity ratios in the exclusive nuclear region were analyzed using ImageJ. The experiment was performed three times and the results were largely the same.

### 4.7. LysoTracker Red Staining

Equal numbers of cells (8 × 104/well) were seeded on glass slides in 12-well plates for 24 h at 37 °C in 5% CO_2_. Then, the cells were incubated for 24 h with the indicated compound. After 24 h, the culture medium was removed and the cells were washed three times with PBS, and stained with LysoTracker Red (37 °C, 5% CO_2_). After 20 min, the cells were washed three times with PBS, and observed under a laser confocal scanning microscope equipped with an argon laser (excitation wavelength 488 nm) and a 63× objective lens, and images were obtained. The red and green intensity ratios in the exclusive nuclear region were analyzed using ImageJ. The experiment was performed three times and the results were largely the same.

### 4.8. Measurement of Intracellular ROS

Cells were prepared and treated as described earlier. Then, the cells were harvested, washed with PBS, and stained with DCFDA (10 μM, D399, Invitrogen, Carlsbad, CA, USA) at 37 °C for 20 min protected from light in a cell incubator. Then, the cells were washed with 37 °C prewarmed PBS, and resuspended in HBSS for detection. Intracellular ROS was measured at the FL1 channel and analyzed with a BD Accuri C6 flow cytometer (BD Pharmingen, San Diego, CA, USA). We performed each independent experiment three times; the mean fluorescence intensity of the cells is expressed as the mean ±standard deviation.

### 4.9. Statistical Analysis

All experiments were repeated at least three times. The *Q* value was derived as follows: *E*_ab_/(*E*_a_ + *E*_b_ − *E*_a_ × *E*_b_), where *E*_a_ and *E*_b_ represent the effects of drugs a and b, respectively, and *E*_ab_ represents the combined effect. If the *Q* value is >1.15, then a and b are synergistic. If the *Q* value is between 0.85 and 1.15, the relation between a and b is additive; a *Q* value of < 0.85 indicates that a and b are antagonistic [49]. The statistical significance of the differences was evaluated using one-way analysis of variance (ANOVA) and multiple comparisons, and the level of significance was set at *p* < 0.001, *p* < 0.01, and *p* < 0.05.

## 5. Conclusions

α-Hed is a novel potent autophagy inhibitor, and combination therapy using α-Hed and Tax is an effective and promising treatment strategy for NSCLC.

## Supplementary Materials

Supplementary materials are available online at http://www.mdpi.com/1422-0067/19/10/3221/s1.

## Abbreviations

NSCLC	non-small cell lung cancer
Tax	paclitaxel
α-Hed	alpha-hederin
SDS-PAGE	sodium dodecyl sulfate–polyacrylamide gel electrophoresis
PBS	phosphate-buffered saline
CatD	cathepsin D
ROS	reactive oxygen species
NAC	N-acetylcysteine
CQ	chloroquine
HCQ	hydroxychloroquine
DMEM	Dulbecco’s modified Eagle’s medium
FBS	fetal bovine serum
HBSS	Hanks’ balanced salt solution
ATCC	American Type Culture Collection
LC3B	microtubule-associated protein light chain 3B
AO	Acridine Orange
Baf	Bafilomycin A1
CCK-8	Cell Counting Kit-8
PARP	poly (ADP-ribose) polymerase
DCFDA	2′,7′-dichlorofluorescein diacetate
