# Supplementary material for: The Novel Autophagy Inhibitor Alpha-Hederin Promoted Paclitaxel Cytotoxicity by Increasing Reactive Oxygen Species Accumulation in Non-Small Cell Lung Cancer Cells
Source: Int J Mol Sci. 2018 Oct 18;19(10):3221. doi: 10.3390/ijms19103221 (PMC6214018; doi:10.3390/ijms19103221)
Supplement: Supplementary file 1 [file ijms-19-03221-s001.pdf]

Additional file

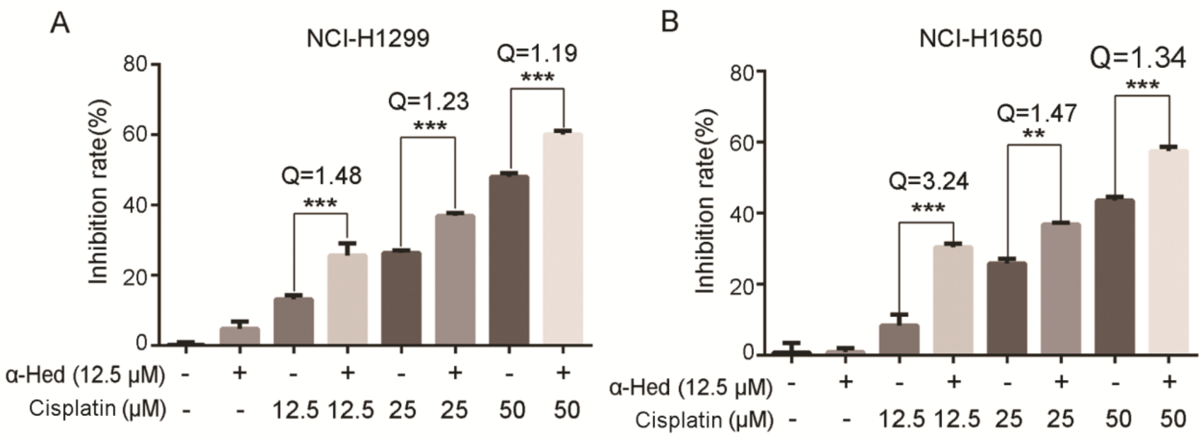

α-Hed increased the cisplatin-induced inhibition of human NSCLC cell proliferation. (a and b) NCI-H1650 (a) and NCI-H1299 (b) cells were treated with or without α-Hed or Tax; 24 h later, the survival rate was detected using CCK-8. $**p < 0.01$, $***p < 0.001$. Q value > 1.15 indicates a synergistic effect.
